# Roles for miRNAs in osteogenic differentiation of bone marrow mesenchymal stem cells

**DOI:** 10.1186/s13287-019-1309-7

**Published:** 2019-06-28

**Authors:** Jicheng Wang, Shizhang Liu, Jingyuan Li, Song Zhao, Zhi Yi

**Affiliations:** 1 Shaanxi Provincial People’s Hospital, 256 Youyi West Road, Beilin, Xi’an, 710068 China; 2 Xi’an Medical University, Xi’an, 710068 China

**Keywords:** Bone marrow mesenchymal stem cells (BMSCs), MicroRNAs (miRNA), Osteogenic differentiation, Bone defects, Bone regeneration, Bone tissue engineering, Treatment

## Abstract

Bone marrow mesenchymal stem cells (BMSCs), which were first discovered in bone marrow, are capable of differentiating into osteoblasts, chondrocytes, fat cells, and even myoblasts, and are considered multipotent cells. As a result of their potential for multipotential differentiation, self-renewal, immune regulation, and other effects, BMSCs have become an important source of seed cells for gene therapy, tissue engineering, cell replacement therapy, and regenerative medicine. MicroRNA (miRNA) is a highly conserved type of endogenous non-protein-encoding RNA of about 19–25 nucleotides in length, whose transcription process is independent of other genes. Generally, miRNA plays roles in regulating cell proliferation, differentiation, apoptosis, and development by binding to the 3′ untranslated region of target mRNAs, whereby they can degrade or induce translational silencing. Although miRNAs play a regulatory role in various metabolic processes, they are not translated into proteins. Several studies have shown that miRNAs play an important role in the osteogenic differentiation of BMSCs. Herein, we describe in-depth studies of roles for miRNAs during the osteogenic differentiation of BMSCs, as they provide new theoretical and experimental rationales for bone tissue engineering and clinical treatment.

## Background

Bone marrow mesenchymal stem cells (BMSCs), which were first discovered in bone marrow by Friedenstein et al. [1], can differentiate into aggregates similar to small-area bone or cartilage. After years of research, it was found that these cells can differentiate into osteoblasts, chondrocytes, adipocytes, and even myoblasts; thus, they are considered multi-potential cells. Indeed, as a result of their potential for multi-directional differentiation, self-renewal, immune regulation, and other effects, BMSCs have become an important source of seed cells in gene therapy, tissue engineering, cell replacement therapy, and regenerative medicine.

MicroRNA (miRNA), a highly conserved type of endogenous non-protein-encoding RNA about 19–25 nucleotides (nt) in length [2], can degrade or induce translational silence by binding to the 3′-untranslated region (3′-UTR) of target mRNAs, thus affecting cell proliferation, differentiation, apoptosis, and ontogeny [3]. Transcription of miRNAs involves a highly conserved process that occurs independently of other genes. Notably, although miRNAs have been implicated in many metabolic processes, they are not translated into proteins [4]. Several studies have shown that miRNAs play an important role in the osteogenic differentiation of BMSCs. We describe in-depth studies of roles for miRNAs during osteogenic differentiation of BMSCs, as they provide new theoretical and experimental bases for bone tissue engineering and clinical treatment.

### Generation and biological function of miRNAs

In 1993, Lee et al. [5] first discovered the presence of small RNAs in *Caenorhabditis elegans* and observed that they controlled biological processes such as the regulation of gene expression [6–8]. Around the same time, Wightman et al. reported the existence of small RNAs such as lin-4 [9]. Reinhart et al. found another small RNA with post-transcriptional regulation in the *C. elegans*: let-7 [10]. With deeper research, more than 1000 miRNAs have since been discovered, each of which regulates multiple mRNAs and is involved in the regulation of biological processes [11].

The production of miRNAs is a very complex biological process that includes two parts, nuclear synthesis and cytoplasmic synthesis, and requires the participation of a variety of enzymes. First, the gene encoding the miRNA is transcribed into a pri-miRNA with special hairpin structures (AAAAA and 7MGpppG) by RNA polymerase II within the nucleus. Next, pri-miRNAs are microcleaved by the nuclease Drosha (ribonuclease III) and processed into miRNA precursors of 70–80 nt with a stem ring structure, i.e., pre-miRNAs. Exportin-5, a cytoplasmic transporter, transports pre-miRNAs from the nucleus into the cytoplasm with the assistance of Ran-GTP, and then the pre-miRNA is cleaved by ribonuclease III (Dicerase) into a duplex structure comprising an miRNA and miRNA* of about 19–23 nt. A miRNA* is the non-miRNA strand of a miRNA duplex generated by a Dicer-like enzyme from the miRNA stem-loop precursor; typically, miRNA*s are degraded. miRNAs form mature miRNAs by binding to argonaute proteins. Subsequently, the guide strand miRNA participates in miRNA transcription, while the passenger strand miRNA is degraded [12]. There are two manners by which mature miRNAs can form an RNA-induced silencing complex (RISC): (1) when the miRNA and target gene are fully complementary, the miRNA degrades the target gene; or (2) when the miRNA and target gene are not fully complementary, the combination of miRNA and 3′UTR inhibits translation of the target gene [13]. miRNAs are involved in several physiological processes, such as development, proliferation, differentiation, and apoptosis of normal cells, as well as in the maintenance of cellular pluripotency [7, 14].

### miRNAs in osteogenic differentiation of BMSCs

**Fig. 1:**
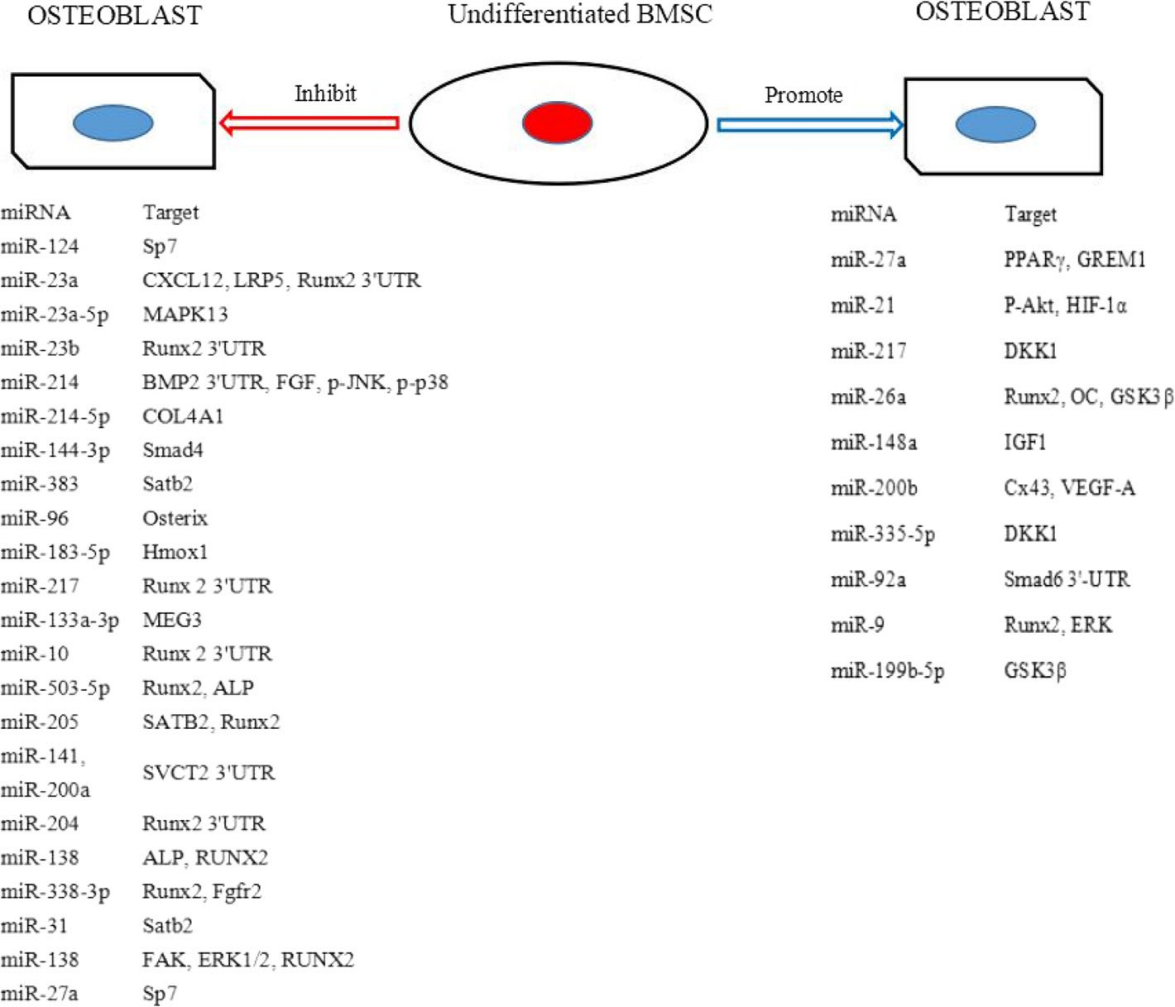
Roles for different miRNAs in osteogenic differentiation of undifferentiated bone marrow mesenchymal stem cells (BMSC)

Osteoblasts, which are involved in bone formation, are differentiated in vivo from BMSCs. Many studies have shown that miRNAs play an important role in osteogenic differentiation of BMSCs, as abnormal miRNA expression had important influences on their osteogenic differentiation [15, 16]. Oskowitz et al. [17] found that after knocking out the mouse endonuclease Dicer, its BMSCs lost osteogenic differentiation. Dicer is an essential endonuclease for miRNA synthesis, indicating that miRNAs are closely related to bone formation and development [18]. Twenty-two differentially expressed miRNAs were identified in bone marrow mesenchymal stem cells (BMSCs) from patients with steroid-induced femoral head necrosis, 17 were upregulated and five were downregulated. During osteogenic differentiation of BMSCs, hsa-mir-601, hsa-mir-452-3p, hsa-mir-647, hsa-mir-516b-5p, and hsa-mir-127-5p were significantly downregulated, while hsa-mir-122-3p was significantly upregulated, suggesting that different miRNAs promoted or inhibited osteogenic differentiation [19]. miRNAs and their identified target genes in the osteogenic differentiation of BMSCs are summarized in Fig. 1.

### Anti-osteogenic differentiation miRNAs

Tang et al. observed a time-dependent decrease in miR-124 expression during osteogenic differentiation of BMSCs, while alkaline phosphatase (ALP) activity and expression of osteocalcin (OCN), osterix (Sp7), and runt-related transcription factor 2 (Runx2) were significantly increased. miR-124 overexpression inhibited osteogenic differentiation of BMSCs, and Sp7 was found to be a direct target [20]. miR-23a was significantly downregulated during osteogenic differentiation of BMSCs on the surface of titanium nanostructures, and its overexpression inhibited osteogenic differentiation of BMSCs; it was revealed that CXC chemokine ligand-12 (CXCL12) was a direct target of miR-23a [21]. miR-23a-5p, another inhibitor, was also significantly downregulated during the osteogenic differentiation of BMSCs; however, upregulation of miR-23a-5p inhibited osteogenic differentiation of hBMSCs by directly targeting mitogen-activated protein kinase 13 (MAPK13) [22]. Similarly, miR-23a was found to be significantly downregulated during osteogenic differentiation of hBMSCs, and its overexpression directly targeted low-density lipoprotein receptor-associated protein 5 (LRP5) to inhibit osteogenic differentiation of hBMSCs [23]. Tian et al. found that overexpressed miR-23a bound to the 3′UTR of Runx2, which reduced its expression level and inhibited osteogenic differentiation of BMSCs. Notably, CXCL13 was found to attenuate the interaction between miRNA-23a and Runx2-3′UTR to promote osteogenic differentiation of BMSCs; thus, it can be used as a novel factor to promote osteogenic differentiation of BMSCs [24]. Deng et al. observed that miR-23b overexpression significantly reduced Runx2 expression levels during osteogenesis by directly binding to the 3′UTR of Runx2 and participating in tumor necrosis factor-α-mediated osteogenic induction of BMSCs. Moreover, injection of Ad-Runx2 attenuated bone loss caused by miR-23b, thus providing a new direction for the treatment of osteoporosis [25].

Several studies have shown that miR-214 is significantly downregulated during osteogenic induction. First, overexpressed miR-214 was shown to bind to the 3′UTR of bone morphogenetic protein 2 (BMP2) to inhibit its expression; whereas, KCNQ1OT1 upregulated BMP2 expression by inhibiting miR-214 to promote osteogenic differentiation of BMSCs [26]. Subsequently, Guo et al. found that overexpression of miR-214 reduced ALP activity and gene expression of OCN, type I collagen (Col I), and osteopontin (OPN), thus inhibiting osteoblast differentiation of BMSCs. miR-214 overexpression was also shown to inhibit protein expression of fibroblast growth factor (FGF), phosphorylated c-Jun N-terminal kinase (p-JNK), and phosphorylated p38 mitogen-activated protein kinase (p-p38) in BMSCs, indicating that miR-214 inhibits osteogenic differentiation of BMSCs by inhibiting JNK and p38 pathways [27]. A later study showed that miR-214-5p inhibited expression of ALP, OCN, Runx2, and collagen alpha-1 (I) chain (COL1A1) in BMSCs during osteogenic differentiation, as well as transforming growth factor-beta (TGF-β), phosphorylated Smad2 (p-Smad2), and collagen IV alpha-1 chain (COL4A1) proteins in BMSCs. Collectively, these results demonstrated that miR-214-5p may attenuate the osteogenic differentiation of BMSCs by regulating COL4A1 [28]. Moreover, the regulation of TGF-β/Smad2/COL4A1 signaling to promote osteogenic differentiation of BMSCs is of great significance for the development of new treatments for postmenopausal osteoporosis.

**Fig. 2:**
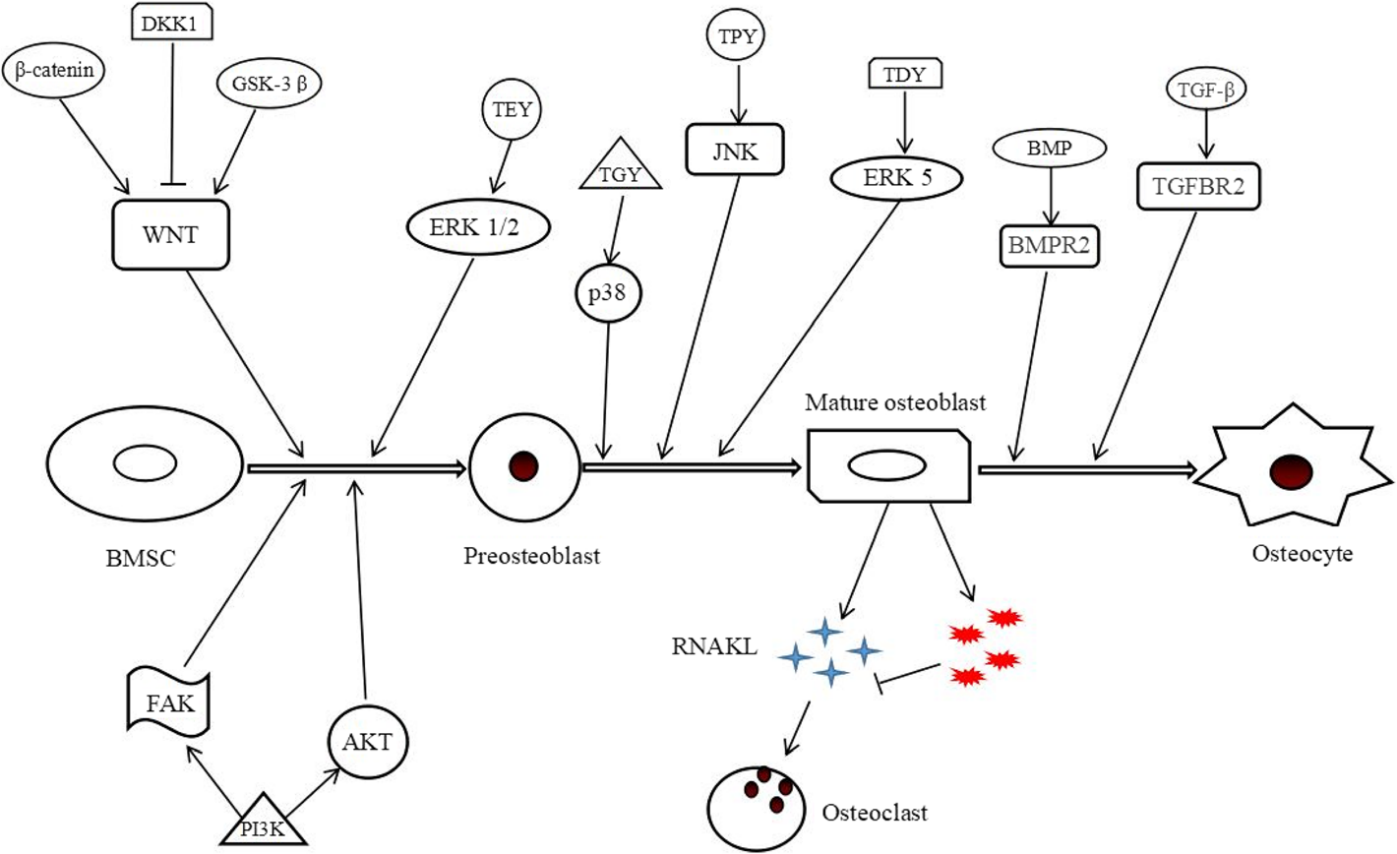
Schematic representation of signaling pathways involved from bone mesenchymal stem cell to osteocyte cell

Lu et al. reported that miR-144-3p inhibits osteogenic differentiation of BMSCs by reducing Smad4 expression [29]. Recent studies found that miR-383 inhibits osteogenic differentiation of rat BMSCs by targeting AT-rich sequence-binding protein 2 (Satb2) [30]. Osterix, a zinc finger-containing transcription factor that plays an important role in osteogenic differentiation and bone formation [31], is also a downstream factor of Runx2, which is a necessary transcription factor for osteogenic differentiation, matrix production, and mineralization during bone formation [32]. Liu et al. observed that miR-96 is significantly upregulated in the serum of elderly patients with osteoporosis, and its overexpression reduced osteogenic differentiation of BMSCs by targeting osterix. Thus, inhibiting miRNA-96 expression can increase osteogenic differentiation of BMSCs, making miR-96 a potential diagnostic marker or therapeutic target for age-related bone loss [33]. Studies have shown that miR-183-5p reduces proliferation and osteogenic differentiation of BMSCs by targeting heme oxygenase-1 (Hmox1), which accelerates aging [34]. Zhu et al. found that miR-217 targeting of the 3′UTR of Runx2 inhibited osteogenic differentiation of BMSCs through extracellular signal-regulated kinase (ERK) and p38 signaling pathways [35]. Upregulation of miR-133a-3p was observed in individuals with postmenopausal osteoporosis, in whom inhibition of miR-133a-3p was subsequently shown to promote osteogenic differentiation of BMSCs and improve symptoms [36]. miR-10 was also shown to inhibit osteogenic differentiation of BMSCs by targeting Runx2 and ERK pathways [37]. Liu et al. found that miR-503-5p overexpression reduced protein expression levels of Runx2 and ALP and inhibited osteogenic differentiation of BMSCs [38]. A summary of how these pathways are involved in the transition of BMSCs into osteocyte cells in shown in Fig. 2.

Hu et al. found that miR-205 binds to Satb2 and Runx2 via ERK and p38 MAPK signaling pathways, whereby it inhibits osteogenic differentiation of BMSCs [39]. Vitamin C is an essential nutrient for bone marrow stromal cell differentiation, collagen synthesis, and bone formation [40–45]. Notably, vitamin C is highly water soluble, but cannot enter the cell by simply diffusing through the lipid bilayer of the plasma membrane; instead, its transport must be mediated by a specific transporter. In BMSCs, vitamin C is transported into cells via sodium-dependent vitamin C transporter 2 (SVCT2) [46]. miR-141 and miR-200a inhibit SVCT2 expression by targeting its 3′UTR, thereby reducing osteogenic differentiation of BMSCs [47]. Zhao et al. showed that overexpressed miR-204 directly bound to the 3′UTR of Runx2, which promoted adipogenic differentiation and inhibited osteogenic differentiation; in contrast, downregulation of miR-204 increased osteogenesis and weakened the adipogenicity of BMSCs [48]. miR-138 was shown to inhibit osteogenic differentiation of BMSCs by targeting ALP and RUNX2. Moreover, delivery of anti-miR-138 to rats significantly enhanced the osteogenic capacity of BMSCs, indicating potentially important clinical significance of anti-miRNA-138 for repair and regeneration of bone defects [49]. Liu et al. showed that overexpression of miR-338-3p can inhibit the expression of osterix by directly targeting Runx2 and Fgfr2, thereby reducing osteoblast differentiation of BMSCs [50]. Deng et al. showed that overexpression of miR-31 could inhibit osteogenic differentiation of BMSCs, while downregulation of miR-31 significantly increased gene and protein expression levels of osteogenic-specific genes in vitro. In addition, injection of anti-miR-31 into rats enhanced the osteogenic differentiation potential and bone-regenerative ability of BMSCs [51]. A subsequent study confirmed that overexpression of miR-31 reduced Satb2 protein expression levels, which attenuated osteogenic differentiation of BMSCs, while downregulation of miR-31 promoted their osteogenic differentiation [52]. In addition, overexpression of miR-138 targets FAK, ERK1/2, and RUNX2 to inhibit osteogenic differentiation of BMSCs [53], which was confirmed in a second study that showed downregulation of miR-138 promotes mechanical tension-induced osteogenesis of hBMSCs [54]. Gong et al. found that overexpression of miR-27a significantly inhibited expression of Sp7, a target gene of miR-27a, and attenuated Satb2-induced osteogenic differentiation of BMSCs [55]. However, a later study found that upregulated miR-27a directly targets peroxisome proliferator-activated receptor gamma (PPARγ) and gremlin 1 (GREM1), which inhibited lipogenesis and promoted osteogenic differentiation of BMSCs [56]. These results indicate that miR-27a can either promote or inhibit osteogenic differentiation of BMSCs by affecting different targets. miRNAs with anti-osteogenic differentiation effects are summarized in Table 1.

**Table 1:** Anti-osteogenic differentiation miRNAs

miRNAs	Target gene/target protein	Expression level	References
miR-124	Sp7	↑	[20]
miR-23a	CXCL12, LRP5, Runx2 3′UTR	↑	[21, 23, 24]
miR-23a-5p	MAPK13	↑	[22]
miR-23b	Runx2 3′UTR	↑	[25]
miR-214	BMP2 3′UTR, FGF, p-JNK, p-p38	↑	[26, 27]
miR-214-5p	COL4A1	↑	[28]
miR-144-3p	Smad4	↑	[29]
miR-383	Satb2	↑	[30]
miR-96	Osterix	↑	[33]
miR-183-5p	Hmox1	↑	[34]
miR-217	Runx 2 3′UTR	↑	[35]
miR-133a-3p	MEG3	↑	[36]
miR-10	Runx 2 3′UTR	↑	[37]
miR-503-5p	Runx2, ALP	↑	[38]
miR-205	SATB2, Runx2	↑	[39]
miR-141, miR-200a	SVCT2 3′UTR	↑	[47]
miR-204	Runx2 3′UTR	↑	[48]
miR-138	ALP, RUNX2	↑	[49]
miR-338-3p	Runx2, Fgfr2	↑	[50]
miR-31	Satb2	↑	[52]
miR-138	FAK, ERK1/2, RUNX2	↑	[53]
miR-27a	Sp7	↑	[55]

### Pro-osteogenic differentiation miRNAs

Although many miRNAs inhibit osteogenic differentiation of BMSCs, exciting results from recent research have revealed miRNAs that promote osteogenic differentiation of BMSCs, including miR-21, miR-217, miR-26a, miR-148a, miR-200b, miR-335-5p, miR-92a, miR-9, and miR-199b-5p.

Yang et al. showed that miR-21 promoted migration and osteogenic differentiation of BMSCs in vitro, as well as their osteogenic capacity, by increasing p-Akt and activating HIF-1α. Moreover, this study confirmed that the BMSC/β-tricalcium phosphate (β-TCP) complex modified by miRNA-21 had a significant osteogenic effect in repairing critical size defects [57]. Another study found that miR-21 was downregulated in bone tissue and serum of patients with osteoporosis. Expression of mineralized nodules and osteogenic genes was significantly increased in SD rats transfected with miR-21 analogues, indicating that miR-21 could promote osteogenic differentiation of BMSCs and, thus, providing a theoretical basis for the development of anti-osteoporosis drugs [58]. Dickkopf-1 (DKK1) is a 29-kDa glycoprotein that plays an important role in maintaining bone metabolism and homeostasis. Abnormal expression of DKK1 is associated with a variety of bone-related diseases, while its inhibition promotes osteoblast differentiation and bone healing [59, 60]. Dai et al. showed that miR-217 promotes nuclear translocation of β-catenin by targeting DKK1, which increases the expression of RUNX2 and COL1A1 to significantly promote proliferation and osteogenic differentiation of BMSCs [61]. This study provides a new approach to the treatment of steroid-associated osteonecrosis.

Repair of bone defects continues to be a major challenge for clinicians because it is difficult to restore bone function and regenerate bone loss. Implantation of BMSCs transfected with miR-26a and β-tricalcium phosphate biomaterials into the defect area of a mouse skull defect model increased bone regeneration and new bone volume, as well as gene and protein expression levels of Runx2 and OCN [62]. Studies have shown that miR-26a targets glycogen synthase kinase three beta (GSK3β) to activate Wnt signaling and promote osteogenic differentiation of BMSCs [63]. Liu et al. found that low expression of miR-148a induces osteogenic differentiation of rat BMSCs by targeting insulin-like growth factor 1 (IGF1), and promotes fracture healing [64]. Subsequently, it was observed that rat BMSCs and human umbilical vein endothelial cells (HUVECs) are functionally connected by connexin 43 (Cx43), and miR-200b can be transferred from BMSCs to HUVECs via Cx43 to regulate osteogenesis and angiogenesis. Moreover, low expression of miR-200b inhibited VEGF-A expression, promoted osteogenic differentiation of BMSCs, and facilitated bone regeneration [65]. Zhang et al. found that overexpression of miR-335-5p induced osteogenic differentiation and bone formation of mouse BMSCs by targeting DKK1, and the resulting modified BMSCs have potential clinical therapeutic value for craniofacial bone regeneration [66]. Another study confirmed that a miR-335-5p lipidoid-miRNA preparation (LMF-335) successfully delivered miR-335-5p into cells and promoted in vitro osteogenesis and in vivo skull bone healing, indicating that lipid-like miRNA delivery can be used to induce osteogenic differentiation of and bone regeneration by BMSCs [67]. Yan et al. reported that miR-92a targeted the 3′-UTR of Smad6 to inhibit Smad6-mediated Runx2 degradation and promote osteogenic differentiation of BMSCs [68]. A later study reported upregulation of miR-9 during osteogenic differentiation of BMSCs, as well as enhancement of their osteogenic capacity by its overexpression, and promotion of osteogenic differentiation through actions on Runx2 and ERK pathways [37]. Similarly, Zhao et al. showed that miR-199b-5p was significantly upregulated during osteogenesis, while its overexpression promoted osteoblast differentiation BMSCs through the GSK3β/β-catenin signaling pathway [69]. miRNAs with pro-osteogenic effects are summarized in Table 2.

**Table 2:** Pro-osteogenic differentiation miRNAs

miRNAs	Target gene/target protein	Expression level	References
miR-27a	PPARγ, GREM1	↑	[56]
miR-21	P-Akt, HIF-1α	↑	[57]
miR-217	DKK1	↑	[61]
miR-26a	Runx2, OC, GSK3β	↑	[62, 63]
miR-148a	IGF1	↓	[64]
miR-200b	Cx43, VEGF-A	↓	[65]
miR-335-5p	DKK1	↑	[66]
miR-92a	Smad6 3′-UTR	↑	[68]
miR-9	Runx2, ERK	↑	[37]
miR-199b-5p	GSK3β	↑	[69]

## Conclusion

Bone marrow mesenchymal stem cells (BMSCs) are an important source of osteogenic seed cells in tissue engineering, and have good prospects for applications in the field of bone defect repair and regeneration. miRNAs play a key role in osteogenic differentiation of BMSCs, but their specific mechanisms of action are not fully understood. Indeed, as different miRNAs promote or inhibit osteogenic differentiation of BMSCs by affecting different targets, further study of the functional specificity of miRNA target genes and interactions between miRNAs is of great significance to elucidate their mechanisms of action. Gene therapy targeting miRNA target genes will benefit patients as research progresses. Moreover, with continuous development of biomedicine, molecular mechanisms underlying osteoblastic differentiation of BMSCs will be increasingly clarified, thus providing new theoretical and experimental rationales for bone tissue engineering and clinical treatment.

## Data Availability

Not applicable.

## Abbreviations

ALP: Alkaline phosphatase
BMP2: Bone morphogenetic protein 2
BMSCs: Bone marrow mesenchymal stem cells
Col I: Type I collagen
COL4A1: Collagen IV alpha-1 chain
Cx43: Connexin 43
CXCL13: CXC chemokine ligand-13
DKK1: Dickkopf-1
ERK: Extracellular signal-regulated kinase
GREM1: Gremlin 1
GSK3β: Glycogen Synthase Kinase 3β
HIF-1α: Hypoxia-inducible factor-1α
Hmox1: Heme oxygenase-1
HUVECs: Human umbilical vein endothelial cells
IGF1: Insulin-like growth factor 1
LRP5: Lipoprotein (LDL)-receptor-associated protein 5
MAPK13: Mitogen-activated protein kinase 13
miRNA: MicroRNA
OC: Osteocalcin
OCN: Osteocalcin
OP: Osteoporosis
OPN: Osteopontin
p38 MAPK: p38 mitogen-activated protein kinase
PMOP: Postmenopausal osteoporosis
PPARγ: Peroxisome proliferator-activated receptor gamma
Runx2: Runt-related transcription factor 2
Satb2: AT-rich sequence-binding protein 2
Smad4: Drosophila mothers against decapentaplegic 4
SONFH: Steroid-induced femoral head necrosis
Sp7: Osterix
SVCT2: Sodium-dependent vitamin C transporter 2
VEGF-A: Vascular epidermal growth factor-A
